# Pelvic angioembolization: how urgently needed?

**DOI:** 10.1007/s00068-020-01510-1

**Published:** 2020-10-09

**Authors:** Giles Lawrence Devaney, Kate Louise King, Zsolt Janos Balogh

**Affiliations:** Division of Surgery, Department of Traumatology, John Hunter Hospital and University of Newcastle, Newcastle, NSW 2310 Australia

**Keywords:** Pelvic fracture, Polytrauma, Angioembolization, Resuscitation

## Abstract

**Purpose:**

Angioembolization (AE) has been questioned as first-line modality for hemorrhage control of pelvic fracture (PF)-associated bleeding due to its potential inconsistent timely availability. We aimed to describe the patterns of AE use with hemostatic resuscitation and hypothesized that time to AE improved during the study period.

**Methods:**

A Level-1 trauma center’s prospective PF database was analyzed. All consecutive PFs referred to angiography between 01/01/2009 and 12/31/2018 were included. All suspected pelvic hemorrhage was managed with AE; pelvic packing was not performed. Demographics, injury/shock severity, 24-h transfusion data, time to AE and mortality were recorded. Data are presented as median (IQR).

**Results:**

During the 10-year study period, 1270 PF patients were treated. Thirty-six (2.8%) [75% male, 49 (33;65) years, ISS 36 (24;43), base deficit 3.65 (5.9;0.6), transfusions 4(2;7)] had AE. The indication for AE was clinical suspicion (CS) of pelvic bleeding [CS 24(67%)] or arterial blush on CT [CT 12 (33%)]. Median time to AE was 141 min for CS, and 223 min for CT, with no change over the study period. Patients with CS had a higher ISS, worse base deficit, greater transfusion requirements and faster time to AE. Five patients (14%) died. There were no deaths attributed to exsanguination.

**Conclusions:**

Time to AE did not improve. Patients referred from CT are physiologically different from CS and should be analyzed accordingly, with CS resulting in faster time to AE in sicker patients. Contemporary resuscitation challenges the need for hyperacute AE as no patients exsanguinated despite time to AE of more than 2 h.

## Background

Hemorrhage control in pelvic fracture-associated bleeding can be difficult to achieve and mortality rates remain high [1, 2]. Pre-peritoneal packing (PPP) and angioembolization (AE) are the most common methods utilized [1, 3]. AE in the context of hemodynamically unstable pelvic injuries has traditionally been considered time critical, with hemorrhage the most common cause of mortality in the first 24 h [4, 5]. Recently, due to the potentially inconsistent timely availability of AE, its role as first-line management for hemorrhage control in pelvic fracture-associated bleeding has been questioned. We aimed to describe the patterns of AE use over the last 10 years and hypothesized that time to AE had improved over the study period.

## Methods

Ethics approval was obtained from the Hunter New England Human Research Ethics Committee.

This study was performed at the John Hunter Hospital (University of Newcastle-affiliated Level-I Trauma Center), New South Wales, Australia. Our institution manages over 4500 trauma admissions per year including approximately 600 patients with an Injury Severity Score (ISS) greater than 12 [6]. Acute pelvic fractures are managed according to the Advanced Trauma Life Support principles as well as our own local pelvic fracture management guidelines [7, 8]. Our local protocol for the management of pelvic fractures begins with judicious hemostatic resuscitation and pelvic binder application by pre-hospital care providers. In the emergency department, initial assessment is conducted which includes early blood gas, trauma series radiography (Chest, Pelvis) and Focused Assessment with Sonography for Trauma. An in-hospital massive transfusion protocol (MTP) can be initiated if clinically required. The MTP is based on Packed Red Blood Cells (PRBC) and Fresh Frozen Plasma (FFP) transfusion and cryoprecipitate in a 1:1:1 ratio, with platelets replacing cryoprecipitate in every even numbered pack and adjustments based on blood gas results, point of care thromboelastography and physiological response. Patients who present with pelvic fractures and hemodynamic instability (with other sources of blood loss addressed according to their priority) are reassessed during the first pack of MTP and if they continue to remain hemodynamically unstable despite hemostatic resuscitation are either transferred directly to angioembolization, or if their hemodynamic response to MTP makes it safe to do so, they are taken for trauma Computed Tomography (CT). At our institution, pre-peritoneal packing is not performed and all suspected pelvic hemorrhage is managed with pelvic angiography, in keeping with current consensus and long-standing functional institutional guidelines [3, 9–11].

Our Trauma Service has maintained a prospective database on all patients presenting with pelvic and acetabular fractures since 2005. This study is a retrospective review of this prospective database over the last 10 years, from January 2009 until January 2019. During this period, our resuscitation protocol was considered a mature unchanged guide for our practitioners. All patients within this database were reviewed for eligibility in this study. All consecutive patients who presented with pelvic fractures within this time period, and were taken to angiography for suspected arterial bleeds, were included. No patients who met inclusion criteria were excluded.

For all patients who met inclusion criteria demographics (age and gender), Injury severity score (ISS), body region-specific Abbreviated Injury Scale (AIS), polytrauma status [defined as AIS > 2 in at least two body regions], hemodynamic instability (defined as physiological parameters prompting the administration of ≥ 2 units of blood products within the initial resuscitation phase), initial shock parameters [systolic blood pressure (SBP), base excess, lactate, hemoglobin, pH] and blood products received (PRBC, FFP, Cryoprecipitate, Platelets) were extracted from the trauma database.

Further information was retrieved from the local electronic patient record system to determine time to angiography for each patient and if angioembolization was successfully performed. The time period was defined as the time between documented arrival time at the hospital and the documented angiography commencement time.

The primary outcome was time to angiography over the study period. Secondary outcomes include transfusion requirements, if embolization was successfully performed, hospital length of stay (LOS), ICU LOS and in-hospital mortality. In an effort to identify possible preventable instances of hemorrhage-related death, all cases of mortality with pelvic fractures from our institutional multidisciplinary death review panel were also assessed.

Patients were categorized into groups according to the indication for angiography, determined as clinically suspected (due to ongoing hemorrhage or hemodynamic instability unresponsive to resuscitation) or due to the presence of arterial blush on CT scanning. This is due to the different physiological presentations of these two cohorts, as patients with a clinically indicated requirement for angiography are considerably more unstable, with worse shock parameters and require greater hemorrhage control.

Continuous data are presented as median with interquartile range, unless otherwise specified. Statistical analysis was performed using linear regression of the outcomes against the patient series when assessing for change in the outcome over time. Comparisons between groups were analyzed using the Independent Samples *T* test, Mann–Whitney *U* test, Fishers Exact test or *X*^2^ test, as appropriate. Significance was established at *p* < 0.05.

## Results

### Patient characteristics

During the study period, 1270 patients presenting with pelvic fractures were treated [48% male, 65 (38–84) years, ISS: 17 (9–24)]. A total of 36 (2.8%) received AE for suspected pelvic vascular compromise. 24 (67%) were based on clinical suspicion (CS) with 12 (33%) referred due to an arterial blush on CT scanning (CT); a complete set of patient characteristics is illustrated in Table 1. Median age of AE patients was 49 (33–65) years with median ISS 36 (24–43.5). Patients who were referred to AE based on clinical suspicion had a greater ISS, were more likely to be hemodynamically unstable, and had a worse base deficit, a higher lactate and lower pH.

**Table 1:** Characteristics of 36 patients receiving angiography

	All patients	CS	CT
Number	36	24	12
% Total	100	67	33
Age	49 (33–65)	49 (33–62)	54 (31–73)
Male %	75	75	75
ISS*	36 (24–43.5)	39.5 (29–46)	24.5 (20–31)
Polytrauma *n* (%)*	28 (78%)	21 (87.5%)	7 (58%)
Hemodynamically unstable *n* (%)*	28 (78%)	24 (100%)	4 (33%)
BD (mmol/L)*	3.65 (5.9–0.6)	5.3 (7.1–3.65)	0.3 (1.2–− 0.7)
Lactate (mmol/L)*	3.55 (2.13–5.25)	3.9 (3.05–6.4)	2.0 (1.25–2.5)
Hb (g/L)	126 (108–142)	125.5 (108–145)	130 (111–137)
pH*	7.28 (7.22–7.32)	7.24 (7.16–7.29)	7.33 (7.31–7.36)
SBP	103 (85–122)	96.5 (85–121)	114 (103–125)

Data are presented as Median with IQR unless otherwise specified

*Indicates *p* < 0.05 based on Independent *T* test, Mann–Whitney *U* test or Chi-Square test (CS vs. CT)

### Time to angiography

**Fig. 1:**
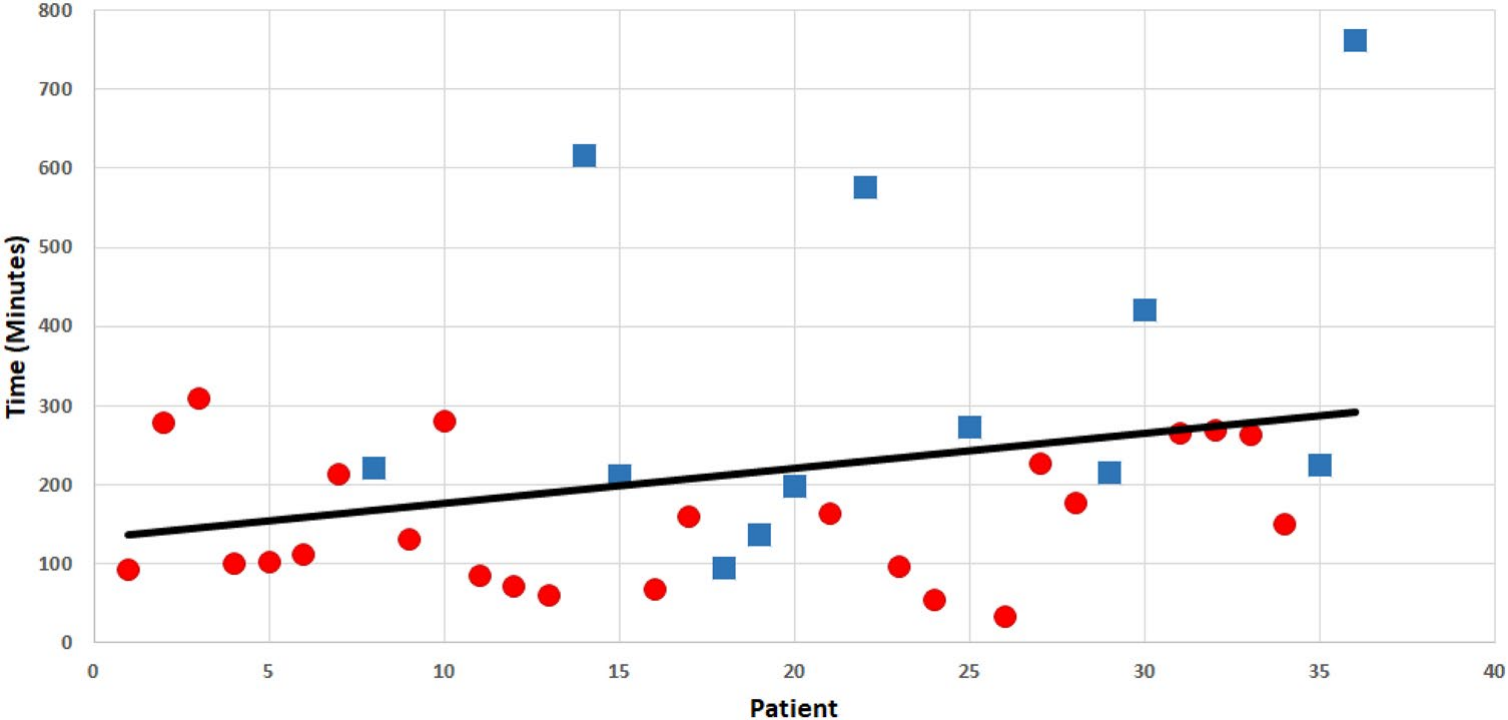
Time to Angiography for 36 patients. Circles indicate CS patients. Squares indicate CT patients

Considering all patients during the study period, the median time to angiography was 187 min (99–266), ranging between 33 and 761 min. The time to angiography for all patients is represented in Fig. 1.

For the overall cohort, linear regression analysis demonstrated a trend towards an increased time to angiography over the study period, however this did not reach significance (*p* = 0.08). The median time to angiography for patients with a clinical suspicion (CS) was 141 (90–236) min, with no change over time (*p* = 0.69). The median time to angiography for patients referred from CT was 233 (208–459) min, with no change over time (*p* = 0.32). AE was utilized in a bimodal fashion, with clinically suspected patients experiencing a faster time to angiography (*p* = 0.011).

### Secondary outcomes

Table 2 summarizes the outcomes of patients referred for AE. 36 patients were taken to angiography for suspected arterial bleeds from pelvic fractures. Of these, 25 (70%) were successfully embolized. Successful embolization was performed in 15 (63%) of patients with a clinical suspicion of pelvic hemorrhage. Despite this relatively low embolization rate, no patients who received angiography died directly from exsanguination, pelvic or otherwise.

**Table 2:** Outcomes of 36 patients receiving angiography

	All patients	CS	CT
Number	36	24	12
Time to AE (min)*	187 (99–266)	141 (90–236)	233 (208–459)
Embolized *n* (%)	25 (70%)	15 (63%)	10 (83%)
Received Transfusion *n* (%)*	28 (78%)	24 (100%)	4 (33%)
PRBC (24 h)*	4 (2–7)	6 (4–10)	0 (0–4)
FFP (24 h)*	3.5 (0–6)	5.5 (3–7)	0 (0–0.5)
Cryoprecipitate (24 h) *	5 (0–10)	10 (5–10)	0 (0–0)
Platelets (24 h)	0 (0–1)	0 (0–1)	0 (0–0)
Hospital LOS*	17.5 (11–30)	26.5 (16–35)	11.5 (10–15)
Required ICU *n* (%)*	26 (72%)	22 (92%)	4 (33%)
ICU LOS	2.5 (0–7)	6 (3–11)	3.5 (2–5)
Mortality *n* (%)	5 (14%)	4 (17%)	1 (6%)

Data are presented as Median with IQR unless otherwise specified

*Indicates *p* < 0.05 based on Independent *T* test, Mann–Whitney *U* test or Chi-Square test (CS vs. CT), as appropriate

Median length of hospital stay for all patients was 17.5 (11–30) days. There was no change in length of stay for the overall cohort, nor within either subgroup. 72% (26/36) of all patients were admitted to the ICU. 22 of the 26 patients admitted to ICU had a clinically indicated cause for angiography, with 92% of this cohort admitted to ICU. The median ICU LOS for patients with a clinical indication for angiography was 6 (3–11) days. There was no change in hospital LOS or ICU LOS for the overall cohort, nor within subgroups.

There were five in-hospital deaths during the study period, representing a mortality rate of 14%. There was no statistically significant change in mortality over time (*p* = 0.94). Causes of death were traumatic brain injury (*n* = 3), Sepsis (*n* = 1) and withdrawal of active care secondary to quality of life concerns by the family (*n* = 1). In the five deceased patients, successful embolization was performed in three, with contrast extravasation not identified for two patients. Four of these patients had a clinical indication for AE, with one patient referred from CT.

In an effort to identify all possible hemorrhage-related pelvic deaths the John Hunter Hospital multidisciplinary death review panel data was analyzed. During the study period, five patients with confirmed or suspected pelvic fractures died in the emergency department. All patients had pre-hospital cardiac arrest with CPR in progress on arrival, with one patient achieving a return of spontaneous circulation before fatally arresting a second time in the emergency department.

### Clinically suspected vs. referred from CT

Baseline patient demographics were significantly different when characterized according to the indication for AE. As demonstrated in Table 1, age and gender were similar between cohorts; however, CS patients had a higher ISS (39.5 vs. 24.5, *p* = 0.005), were more likely to be classified as polytrauma (87.5% vs. 58%, *p* = 0.047), were more likely to be hemodynamically unstable (100% vs. 33%, *p* < 0.001), had a worse base deficit (5.3 vs. 0.3, *p* < 0.001), a higher lactate (3.9 vs. 2.0, *p* < 0.001) and were more acidotic (pH 7.24 vs. 7.33, *p* < 0.001). The transfusion requirements and outcomes also differed between groups. As outlined in Table 2, CS patients had a faster time to AE (141 vs. 233 min, *p* = 0.011), were more likely to require a blood transfusion (100% vs. 33%, *p* < 0.001), required greater amounts of PRBC (6 vs. 0, *p* < 0.001), FFP (5.5 vs. 0, *p* < 0.001) and cryoprecipitate (10 vs. 0, *p* < 0.001). CS patients had a longer hospital admission (26.5 vs. 11.5 days, *p* = 0.022) and were more likely to require ICU level care (92% vs. 33%, *p* < 0.001).

## Discussion

Timely identification and control of pelvic hemorrhage is essential to prevent pelvic fracture-related mortality. AE is widely used for hemorrhage control in arterially bleeding pelvic fractures; a group of patients representing up to 15% of major pelvic fractures and associated with higher mortality than all pelvic fractures [12, 13].

Recently, the usefulness of AE in management of acutely hemorrhaging patients has been questioned due to its potentially inconsistent timely availability. There are many factors that contribute to timely AE, most important being the availability of the interventional radiologists and procedure team to perform the operation. Many studies have investigated the impact that delayed angiography has on patient outcomes. A recent 2-year retrospective cohort study by Matsushima et al. reported an increased in-hospital mortality for patients with increased time to AE, but no increase in 24-h mortality or difference in mortality between time-stratified groups [14]. An earlier landmark paper by Agolini et al. reported a substantial increase in survival when embolization is performed within 3 h of arrival, 36% vs. 75% [9]. Multiple other studies have investigated the impact of AE on patient mortality with varying conclusions. Tanizaki et al. used a smaller cut-off and reported significant increases in mortality for patients who received AE beyond 60 min, a comparative mortality rate of 16% vs. 64% [15]. Conversely, Tesoriero et al. performed a 10-year retrospective review finding no association between time to AE and mortality, with an overall mortality rate of 18% [16].

The patients in our current study who received angiography for pelvic hemorrhage had a median time to AE of 187 (99–266) min. This is well beyond the desirable optimal standard in our institution for achieving hemorrhage control in an acutely bleeding trauma patient [8, 17]. Despite this delayed time to AE, the mortality rate remained low (14%) and unrelated to pelvic bleeding compared to other studies. We attribute this low mortality rate to the early protocol-driven hemostatic resuscitation our institution can provide, as described above. With this strategy, in cases with bound pelvic ring and early administration of clotting factors, venous bleeding appears to no longer be a clinical problem. The arterial bleeders also seem to be less catastrophic than used to be 20 years ago [18], when crystalloid and PRBC-based resuscitation targeting normal systolic blood pressure was the norm [19].

Advancements in resuscitation and stabilization techniques have had a major impact on the modern management of hemodynamically unstable pelvic fractures. Gaski et al. recently reviewed the outcomes of patients at their hospital before and after the institutional adoption of damage control principles and massive hemorrhage control protocols [20]. They identified both a decreased RBC transfusion requirement, decreased need for AE or extra-peritoneal packing and a significant decrease in hemorrhage-related death. The decreased requirement for interventional hemorrhage control was attributed to improved resuscitation strategies, similar to what our institution provides. The transfusion requirements, injury severity and overall mortality rates of the patients in the post-protocol cohort were similar to the patients in our study; however, our patients had a longer time to AE yet recorded no hemorrhage-related deaths.

The low mortality rate and lack of hemorrhage-related deaths, despite median time to angiography of > 3 h, demonstrates the significant impact modern hemostatic resuscitation strategies have on the management of hemodynamically unstable pelvic fractures. This data therefore questions if, with modern resuscitation techniques, hyperacute AE is as necessary as previously believed.

Whilst this study is based on prospectively collected data, the authors acknowledge the study is limited by its retrospective nature. However, we believe the availability of our trauma registry’s independent prospective pelvic fracture database provides better quality evidence than most retrospective studies, as it allowed all consecutive patients presenting with pelvic fractures to be screened for inclusion in this study. Unfortunately, this database does not prospectively collect coagulation data. Information on coagulation factors and time to coagulopathy was therefore not assessed as the authors believe retrospective collection of coagulation data is extremely inaccurate. Further limitation of this work is the non-inclusion of pre-hospital deaths from pelvic hemorrhage. We did identify all patients with significant hemorrhage of possible pelvic origin who died in the emergency department before angiography could be performed. It is important to note that these data are all from a single, well-resourced, Level-1 trauma center with extensive experience in the management of critically injured patients and consequently, outcomes may not be reproducible in hospitals without these resources or experience. The authors acknowledge that this is a relatively small, single-center study, and further research is required.

## Conclusion

Time to AE did not improve during the 10-year study period. AE is utilized in a bimodal fashion, with clinical suspicion resulting in faster time to AE. This paper highlights that contemporary resuscitation techniques challenge the need for hyperacute AE as, throughout the 10-year period, no patients who presented with pelvic fractures died from exsanguination, despite a median time of over 2 h for patients with clinical indication requiring AE. It is also clear from this data that patients referred for AE due to a clinical indication are physiologically very different to patients referred from CT and the authors suggest that future papers report these cohorts separately for more accurate analysis.

## Data Availability

De-identified data can be available upon request.
